# Targeted Alpha Therapy Approach to the Management of Pancreatic Cancer

**DOI:** 10.3390/cancers3021821

**Published:** 2011-04-01

**Authors:** Barry J. Allen, Syed M. Abbas Rizvi, Chang F. Qu, Ross C. Smith

**Affiliations:** 1 Centre for Experimental Radiation Oncology, St George Cancer Care Centre, Gray St, Kogarah, 2217, Australia; E-Mails: syed.rizvi@sesiahs.health.nsw.gov.au (S.M.R.); changfa.qu@gmail.com (C.F.Q.); 2 Cancer Surgery Laboratory, Northern Clinical School, University of Sydney, Kolling Institute, Royal North Shore Hospital, St. Leonards, NSW 2065 Australia; E-Mail: ross.smith@sydney.edu.au (R.C.S.)

**Keywords:** pancreatic cancer, C595, PAI2, *in vitro*, *in vivo*, MUC1 and uPAR tumor expression, TAVAT

## Abstract

Evidence for the efficacy of targeted alpha therapy for the control of pancreatic cancer in preclinical models is reviewed. Results are given for *in vitro* pancreatic cancer cells and clusters and micro-metastatic cancer lesions *in vivo*. Two complementary targeting vectors are examined. These are the C595 monoclonal antibody that targets the MUC1 antigen and the PAI2 ligand that targets the uPA receptor. The expression of the tumor-associated antigen MUC-1 and the uPA receptor on three pancreatic cancer cell lines is reported for cell clusters, human mouse xenografts and lymph node metastases, as well as for human pancreatic cancer tissues, using immuno-histochemistry, confocal microscopy and flow cytometry. The targeting vectors C595 and PAI2 were labeled with the alpha emitting radioisotope ^213^Bi using the chelators cDTPA and CHX-A″ to form the alpha-conjugates (AC). Cell clusters were incubated with the AC and examined at 48 hours. Apoptosis was documented using the TUNEL assay. *In vivo*, the anti-proliferative effect for tumors was tested at two days post-subcutaneous cell inoculation. Mice were injected with different concentrations of AC by local or systemic administration. Changes in tumor progression were assessed by tumor size. MUC-1 and uPA are strongly expressed on CFPAC-1, PANC-1 and moderate expression was found CAPAN-1 cell clusters and tumor xenografts. The ACs can target pancreatic cells and regress cell clusters (∼100 μm diameter), causing apoptosis in some 70–90 % of cells. At two days post-cell inoculation in mice, a single local injection of 74 MBq/kg of AC causes complete inhibition of tumor growth. Systemic injections of 111, 222 and 333 MBq/kg of alpha-conjugate caused significant tumor growth delay in a dose dependent manner after 16 weeks, compared with the non-specific control at 333 MBq/kg. Cytotoxicity was assessed by the MTS and TUNEL assays. The C595 and PAI2-alpha conjugates are indicated for the treatment of micro-metastatic pancreatic cancer with over-expression of MUC1 and uPA receptors in post-surgical patients with minimal residual disease. The observation of tumor regression in a Phase I clinical trial of targeted alpha therapy for metastatic melanoma indicates that alpha therapy can regress tumors by a process called tumor anti-vascular alpha therapy (TAVAT). As a consequence, this therapy could be indicated for the management of non-surgical pancreatic cancer tumors.

## 1. Introduction

The most recent estimates for the incidence of pancreatic cancer in the United States for 2010 (American Cancer Society) are about 43,140 men and women in equal numbers will be diagnosed with pancreatic cancer. Of these, some 36,800 men and women will die of pancreatic cancer. Over the past 15 to 25 years, rates of pancreatic cancer have dropped slightly but pancreatic cancer remains the fourth leading cause of cancer death overall. The lifetime risk of developing pancreatic cancer is about one in 72 (1.4%) and is similar for both men and women.

Advanced pancreatic cancer is associated with a very poor prognosis although surgical resection or radiotherapy is potentially curative for localized disease. The major failure is the late detection of the disease and in the management of metastatic cancer that results in the five year survival for patients being less than 5% [1]. Multimodality treatments, including surgery, chemotherapy, and post-operative radiation therapy, have resulted in only an incremental increase in survival [2]. Novel therapeutic approaches are urgently needed that can kill cancer cells in transit or lesions at the preangiogenic stage, as may be present in minimal residual disease (MRD), as well as regress advanced tumors and so change the course of the disease.

One potential approach is the use of radiolabeled antibodies that are able to target metastatic as well as primary tumor sites with minimal reactivity to normal tissues. Radiolabeled antibodies have been used with some success in different cancers [3].

Targeted alpha-particle therapy (TAT) uses a targeting vector (antibody or ligand) labeled with an alpha emitting radioisotope. The short ranger and high rate of energy loss of the alpha radiation is toxic to targeted cells but not normal cells if outside the 20–80 μm alpha particle range. TAT is in clinical trial for single-cell disorders such as leukemia and micrometastases of carcinomas, in which rapid targeting of cancer cells is possible [4–6]. TAT offers the potential to inhibit the growth of micrometastases by selectively killing isolated and pre-angiogenic clusters of cancer cells in a way not possible for beta radiation.

The most effective radiation treatments are those that not only target the cancer cells but also cause the greatest amount of lethal or non-repairable damage to DNA. Alpha particles with their high linear energy transfer are most effective in this respect. A large number of *in vitro* and *in vivo* experiments with alpha-immunotherapy have shown dramatic superiority over beta-immunotherapy, as only a few alpha hits of the nucleus are needed to achieve cell kill [6–11]. Clinical trials are in progress for, *in alia*, acute myelogenous leukaemia [12] and metastatic melanoma [13,14].

A new concept in alpha therapy, called tumor anti-vascular alpha therapy (TAVAT), has the potential to regress tumors by destroying the tumor capillaries and starving the tumor cells of oxygen and nutrients essential for not only growth but survival of cancer cells as well [15]. TAVAT depends primarily on the permeability of neogenic tumor capillaries.

The paper reviews the expression of the generic receptors MUC1 and uPAR by selected pancreatic cancer cell lines, human tumor xenografts and pancreatic cancer tumors in patients. These receptors are targeted by the vectors C595 and PAI2 respectively. While benign in themselves, these vectors become highly cytotoxic agents when labeled with an alpha emitting radioisotope Bi-213 [16–18]. These alpha therapy agents are the only ones applied so far to pancreatic cancer. As such, this review compares results obtained with these two agents, providing a rationale to proceed to a Phase I clinical trial for the preferred alpha conjugate.

## 2. Targeting Vectors

### 2.1. C595

MUC1 is a well-documented example of a marker that influences pathophysiological behavior. High molecular weight glycoproteins, described as mucins or mucin-like glycoproteins, are frequently found associated with breast carcinoma and other epithelial cell adenocarcinomas [19]. Cancer associated MUC1 is structurally different from normal MUC1 in that the former has shorter and less dense *O*-glycan chains, exposing novel regions of the protein core. The large extracellular domain is dominated by a heavily *O*-glycosylated region consisting of a variable number of 20 amino-acid tandem repeats (VNTRs) [20]. The number of these VNTR sequences is subject to genetic polymorphism, varying between 30 and over 100.

MUC1 function involves mediating cellular transformation in integrating the growth factor receptor and Wnt signaling pathways [21]. MUC1 expression causes anchorage independent growth and tumor formation and is a useful marker for the prognosis of the patients with carcinoma. Patients with MUC1 expression in the carcinoma show significantly lower survival rates than those without MUC1 expression [22,23].

MAb C595 (also known as NCRC48) is reactive with the protein core of MUC1 mucin. The target epitope of the MAb C595 is the tetrameric motif Arg-Pro-Ala-Pro that is repeated many times within the MUC1 protein core [20,24]. The reactivity of the MAb C595 with synthetic peptides containing this motif permits efficient antibody purification using peptide epitope affinity chromatography, which, unlike other methodologies, enables exclusion recovery of functionally active antibody.

The target epitope of the C595 is the tetrameric motif Arg-Pro-Ala-Pro that is repeated many times within the MUC1 protein core [24]. Epithelial cancers often overexpress MUC1, and the overexpression is associated with poor survival [25].

### 2.2. PAI2

An alternative approach to monoclonal antibody (MAb) targeting is the urokinase plasminogen activator (uPA) system, which plays an important role in tumor growth and metastasis [26–28]. These findings suggest that the uPA system is causally involved in cancer progression. Given the poor prognosis of pancreatic cancer patients it is not surprising that there was increased expression of uPA and uPAR in fresh pancreatic cancer tissue and that over expression was related with poorer survival [29]. That uPA and its receptor uPAR can act as a target for anti-cancer therapy has been extensively documented in mouse models [30,31]. Bound to its specific cell surface receptor (uPAR), uPA efficiently converts the inactive zymogen, plasminogen, into the active serine protease, plasmin, which then cleaves either directly or indirectly extracellular matrix components including laminin, fibronectin, and collagen [26,27]. uPAR was initially identified as the cellular binding site for uPA, and the structural basis for this interaction has now been studied extensively. While the uPA/uPAR binding determinants within uPA have been mapped to a single epitope within the amino terminal growth factor like domain, the regions of uPAR critical for binding are multiple and may involve a conformational change of the receptor. The impressive prevalence of over-expression of uPAR and uPA in malignant human cancers, together with the fact that the uPA/uPAR system is not essential for fertility or survival under physiological conditions, makes targeting of this system by recombinant inhibitors very promising.

The activity of uPA is physically regulated by plasminogen activator inhibitors type 1 and 2 (PAI1 and PAI2) [28,32]. PAI2 (MW = 47 kD) is a member of the serine protease inhibitor (Serpin) superfamily and forms SDS-stable 1:1 complexes with uPA. PAI2 can inhibit cancer cell invasion and metastasis [32]. Exogenously added PAI2 inhibited the plasminogen-dependent degradation by cancer cells of the basement membrane components collagen, proteoglycan, and glycoprotein [33].

The median and range of the ratios of uPA mRNA measures between tumor tissue and non-involved pancreatic tissue is 17.1 (1.4–653.6) for pancreatic adenocarcinoma (*P* < 0.001), 3.9 (0.7–7.7), for ampullary carcinoma (*P* = 0.055) and 1.9 (0.6–5.9) for mucinous cystadenoma tissue (*P* = 0.052) [34]. Tumors with low uPA are associated with an exuberant stromal reaction, whereas uPA high tumors show little stromal response. Immunohistochemistry confirms that uPA protein is more prevalent in pancreatic adenocarcinoma tissue than in normal tissue and that it is membrane-bound. uPA mRNA expression is significantly associated with poorly differentiated pancreatic cancers (*P* < 0.05) and positively associated with tumor stage. Tumors which express high amounts of PAI2 which inhibits the uPA/uPAR interaction have significantly improved survival [35]. It is particularly interesting that when uPAR expression was inhibited by interfering RNAs in pancreatic cancer cell lines there was reduced proliferation and mobility with an increase of apoptosis which appeared to involve the ERK signaling pathway [36]. These reports suggest that treatments targeted to the uPAR protein may influence survival of patients with pancreatic cancer.

The targeting characteristics of ^213^Bi-PAI2 allow the alpha radiation to deliver a large fraction of the total decay energy to the nucleus of those cancer cells with high expression of uPA/uPAR. Thus the most malignant pancreatic cancer cells receive the highest radiation dose, with greatly reduced irradiation of distant normal cells. Cell killing occurs after the uPA/uPAR-^213^Bi-PAI2 complex formation with the decay of ^213^Bi and is most effective on endocytosis of the complex, which occurs in 40 minutes [37].

These observations suggest that significant over expression of uPA correlates closely with the rapid progression and invasiveness of pancreatic cancer and that uPA may provide a therapeutic target for pancreatic cancer treatment.

## 3. Methods

### 3.1. Monoclonal Antibodies

C595 MAb was obtained from Nottingham University, U.K.; now available from Medical Scitec Australia Pty Ltd., NSW, Australia. Mouse anti-human aMOPC IgG1 MAb (known as A2) was used as non-specific control. Human recombinant PAI2 (47 kD) was provided by Network Pty Ltd, NSW, Australia; now PAI2 Pty Ltd. Mouse anti-human uPA IgG antibody (#394) was purchased from American Diagnostica Inc (Greenwich, CT, USA).

The chelator cDTPA was purchased from Aldrich Pty Ltd, Australia and DTPA-CHX-A″ was obtained from NIH, Bethesda, USA. Other details are listed in the referred papers.

### 3.2. Preparation of Alpha Conjugates

The alpha particle emitting radionuclide Bi-213 was produced from the Ac-225:Bi-213 generator system, produced by the Institute for Transuranium Elements (ITU), Karlsruhe, Germany [38]. Bi-213 was eluted, presumably as (BiI_5_)^2−^ anion species, from the Ac-225: Bi-213 generator with 1 mL of freshly prepared 0.15 M distilled, stabilized hydriodic acid followed by washing with 250 mL water, and neutralized to pH 5.5 via the addition of 85 mL of DPBS and 0.5 M citric buffer 65 mL (pH 5.5). A time of 2–3 h was allowed for Bi to grow back in the generator for the next elution.

The cDTPA or CHX-A″ chelators were first bound to the targeting vectors C595 and PAI2 and the non-specific controls BSA and A2 and then used to chelate the Bi-213. The alpha-specific activity of the conjugates was 100 kBq: 1 mg. Radiolabeling and purification of protein constructs with Bi were carried out using published methods [39]. Removal of unbound Bi-213 via PD-10 gel filtration columns improved the purity of the Bi-conjugate. The radiolabeling efficiency was determined by Instant Thin Layer Chromatography (ITLC) using a 10 mL aliquot of the final reaction mixture applied to Gelman paper (strip size 1–9 cm, Gelman Science, Ann Arbor, MI). The paper strips, using 0.5 M sodium acetate (pH 5.5) as the solvent, were cut into four sections and the 440 keV gamma emissions from Bi-213 in each section were counted using a 340–540 keV gamma ray window. The radiolabeled protein was found at the origin, while free radioisotope was found at the solvent front. The Bi labeling efficiency for C595, PAI2 and BSA was 90–95%.

### 3.3. Cell Lines and Spheroid Cell Cultures

Three human pancreatic cancer cell lines, CFPAC-1, PANC-1 and CAPAN-1 were obtained from American Type Culture Collection (ATCC, Manassas, VA 20108 USA). The medium and monolayer cell culture used have been described previously [16].

As *in vitro* multi-cell spheroids (MCS) resemble micrometastases during the avascular phase of tumor development, this model has applications for evaluation of the efficacy of radio-immunotherapy for micrometastases [40–44]. Spheroids can also resemble the *in vivo* situation with regard to cell shape and cellular environment, which in turn can determine gene expression and the biological behavior of the cells [45]. MCSs were used as an *in vitro* model of micrometastases of pancreatic cancer and the MUC1 expression determined [17].

CFPAC-1, PANC-1 and CAPAN-1 cells in a monolayer culture were trypsinized and approximately 10^6^ cells in 1.5 mL of complete medium (as used for monolayer cultures) were transferred into each of the 24 wells in a multi-well plate coated with 5% agarose. Cells were aggregated in the multi-wells on a slowly rotating platform within an incubator in 5% CO_2_. Two days after aggregation was initiated, the selected spheroids were examined under an inverted phase-contrast microscope with an ocular scale using an Eppendorf pipette, and transferred into 6 well plates coated with 5% agarose containing 6 mL of complete medium supplemented with FBS in 5% CO2. The medium was changed every two days.

The size and number of spheroids were determined using an inverted phase contrast microscope. Spheroid volume (V) was calculated from the geometric mean of the perpendicular diameters D = (D_max_ + D_mi_n)/2, (V = 4/3π (D/2)^3^). Complete dissociation of the spheroids was achieved by incubation in 0.25% trypsin-0.05% EDTA and PBS (Ca and Mg free) at 37 °C for 15 min, followed by mixing the suspension with a pipette, so as to count the cell numbers in the spheroid. Cold medium was added to stop trypsin action [46]. There are about 500 cells in a 100 μm diameter cell cluster of pancreatic cancer cells.

### 3.4. Immunohistochemistry

The indirect conjugated peroxidase method was used to detect the expression of MUC1 receptor on pancreatic cancer spheroids [11]. Slides for spheroids were fixed for 20 min at RT in 4% paraformaldehyde (1 × PBS, pH 7.4) or for 10 minutes at RT in acetone.

The Northern Sydney Health Human Research Ethics Committee gave approval for informed consent to be obtained from 53 patients undergoing pancreatic resection for a pancreatic cancer mass at Royal North Shore Hospital, NSW, Australia for MUC1 immunohistochemistry. A second batch of 30 patients (male 16, female 14, range 49–81 years, average age 69.7 years) were approved for uPAR testing. Six matched lymph node metastases and ten normal pancreatic tissues from patients were also obtained.

Tumors were fixed in 10% neutral buffered formalin. Paraffin sections (5 μm) were cut from the paraffin embedded blocks, deparaffinized in xylene, following by a graded series of ethanol (100%, 95% and 75%) and rehydrated in tris-buffer saline (TBS, pH 7.5). The sections were incubated with the primary antibody at 13.0 μg/mL (for pancreatic subcutaneous xenograft models) or at 6.5 μg/mL (for cell clusters) at 4 °C for 12 h. Following washing with TBS, slides were incubated with HRP conjugated rabbit anti-mouse IgG (6.5 μg/mL) for 45 min at RT and then washed with TBS 2 times, and developed with diamino benzoate (DAB) substrate solution for 5–10 min at RT. The primary antibody was C595 for MUC1 and #394 for uPAR. The criteria for assessment were described previously [16].

### 3.5. TUNEL Assay

This assay was performed to investigate the lethal pathway after treatment with the ACs. The pancreatic cancer cell clusters were cultured in 6 well plates and incubated in medium as described above. 100 μm (n = 20) cultured cancer cell clusters were treated with 370 kBq of ^213^Bi-C595 AC (test) and controls including 10 μg of C595 alone, 370 kBq ^213^Bi alone and the same activity of non-specific control AC 370 kBq ^213^Bi-A2 at 37 °C. Morphological change was observed at 48 h post-treatment. Apoptotic cells were detected using the TUNEL method [47]. The exposed 3′-OH ends of DNA fragments generated by apoptotic DNA cleavage were detected by TUNEL assay, in which the non-apoptotic cells stained green while apoptotic cells stained brown.

### 3.6. In Vivo Treatment Protocols

Female 6–8 week old BALB/c (nu/nu) athymic nude mice were obtained from the Animal Resources Centre (ARC), Western Australia. Mice subcutaneous (sc) tumor model were established by inoculation of 2 × 10^6^ CFPAC-1 cells, suspended in 200 μL of the serum free medium, using a 29G (0.33 mm × 12.7 mm) needle with a 0.5 mL syringe, into the sc space of the right flank region. Tumor size was documented by measurements using vernier calipers. Tumor volumes were calculated by the following formula: length × width × height × 0.52 in millimeters [48]. Mice were euthanized by cervical dislocation while under anesthesia if tumor size exceeded 10 mm in any dimension.

The dose effect of the AC was examined in mice. Groups of five mice received an IP injection of 222, 296 and 370 MBq/kg of C595-AC and bodyweight was compared with untreated control mice [17]. In the case of the PAI2-AC, the acute MTD > 1420 MBq/kg was found up to 13 weeks [52]. This was extended to 40 weeks [49]. Mice were euthanased by cervical dislocation under anesthesia if they had lost >20% bodyweight, suffered distress or survived to 40 weeks. Rabbits were also injected and monitored for 13 weeks. Blood and organs were collected for pathology. The effect of lysine protection to limit renal uptake of PAI2 was also studied.

Efficacy was studied for local and systemic administration of the C595-AC [17]. Local injection of the AC was made at the inoculation site at two days post-CFPAC-1 cell inoculation. Five groups of five mice each received 1.85, 3.7 and 7.4 MBq of AC (treatment groups), ^213^Bi-A2 at 7.4 MBq (nonspecific control) or cold control mix (non-labeled chelated conjugate). The experiment was terminated after 16 weeks, mice were sacrificed under anesthesia and tumors were excised for histology.

Systemic efficacy was studied by intraperitoneal injection at two days post-CFPAC-1 cell inoculation. Groups of five mice each received 111, 222 and 333 MBq/kg of AC, ^213^Bi-A2 at 333 MBq/kg or cold conjugate control mix. After 16 weeks, the experiment was terminated and tumor xenografts from sacrificed mice were immediately fixed in 10% neutral buffered formalin for MUC-1 expression and H&E staining in paraffin sections. The endpoints were tumor growth to 10 mm in any axis.

## 4. Results

### 4.1. Expression of MUC1 and uPAR in Pancreatic Cancer

#### 4.1.1. MUC1

**Figure 1.**
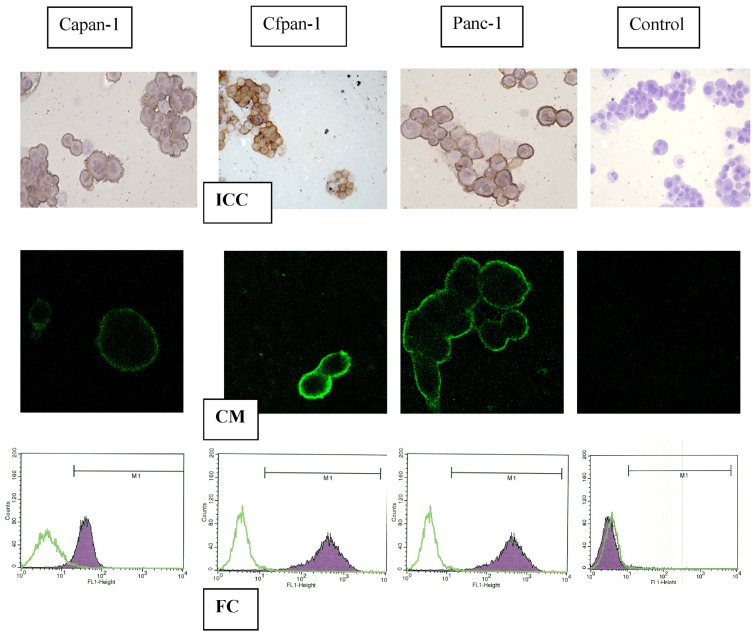
Flow cytometry (FC), confocal microscopy (CM) and immuno-cytochemistry (ICC) after incubation in C595 in three pancreatic cancer cell lines, compared with controls [16].

The immunoreactivity of pancreatic cancer cell lines to C595 is shown in Figure 1. Strong MUC-1 expression (brown stain) was found in each case (A,B,C) but not for the A2 control (D,E,F). MUC1 cell membrane expression was confirmed by confocal microscopy (G,H,I) (green image) and by flow cytometry (J,K,L) (purple peak). These results are scored with those from flow cytometry, confocal microscopy and cell survival from incubation in the AC (Table 1 and Table 2).

#### 4.1.2. uPAR

**Figure 2.**
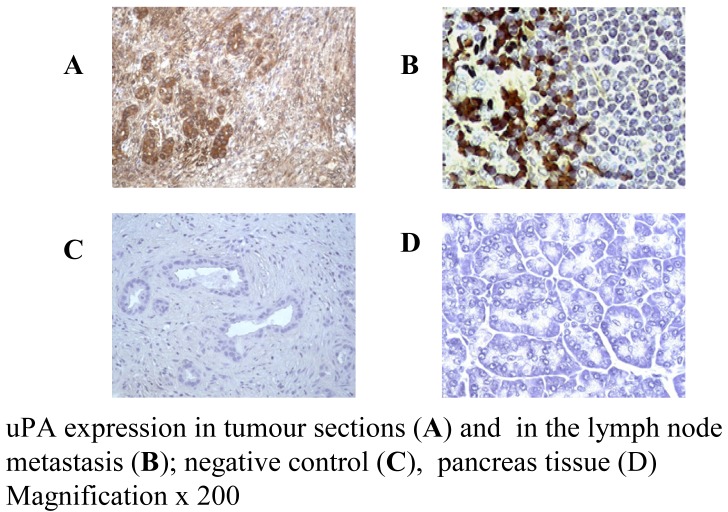
uPA is expressed by pancreatic cancer tumours (**A**,**B**) but not normal tissue (**D**) [18].

Expression of uPAR in pancreatic cancer cell lines was identified by using MAb #394 (anti-uPAR). Immunocytochemistry (ICC) staining of CFPAC-1, CAPAN-1 and PANC-1 cell lines was strong positive for uPAR, but negative for isotype control or omitted primary MAb (Table 2). These results indicate that a high expression of uPA/uPAR was found in both metastatic and primary pancreatic cancer cell lines, as shown in Figure 2.

#### 4.1.3. Human Tumor Tissues

The staining intensity was scaled from positive (+) to strong positive staining (+++) and over-expression of markers is defined as being ++ or +++. Pancreatic cancer tissues over-express MUC1 in 81% (43/53) of patient tumors. uPAR is over-expressed in 87% (26/30) of tumors and 100% (6/6) of matched lymph node metastases, while no immunoreactivity was found with isotype control and in normal pancreas tissue. Results are summarized in Table 1 and Table 2, where normal pancreas expression is essentially negative.

**Figure 3.**
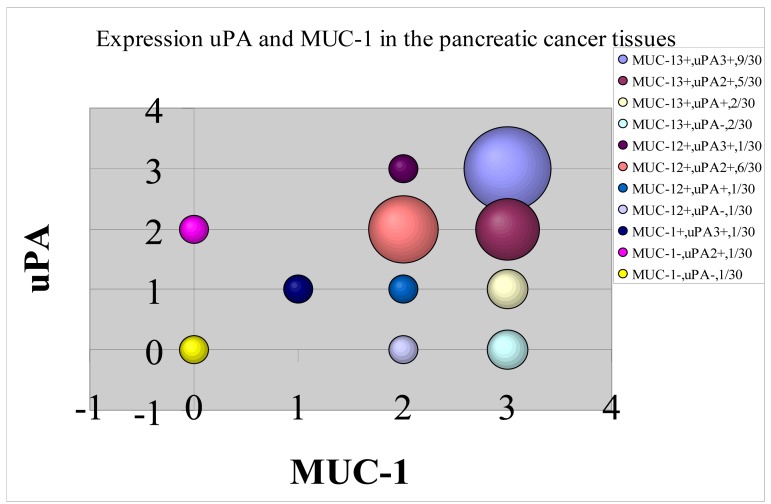
Correlation between the expression of the MUC1 and uPA receptors.

The above results show that both MUC1 and uPA are strongly expressed in the three pancreatic cancer cell lines and in most pancreatic tumors from patients. While these receptors are completely different in nature, the question is whether their expression is analogous or correlated, or complementary and uncorrelated. The answer is given in Figure 3 where a high degree of correlation is observed. The size of the circle represents the number of events. While there are some results for which uPA and MUC1 are highly uncorrelated (zero *vs.* 2+), the great majority of events show a high correlation in expression in the 2+ and 3+ data.

### 4.2. Effects of Targeted Alpha Irradiations

*In vitro* incubation of pancreatic cancer cells in the ACs shows pronounced effects. As shown in Table 2, the control incubations (with non-specific A2) gave very small D_0_ values compared with those for the C595 and PAI2 targeting vectors; the therapeutic D_0_ ratio being ∼15.

CFPAC-1 cultured cell clusters were incubated with C595-AC, showing morphological changes, *i.e.*, clusters dissociated and cells became smaller and rounded. Complete disaggregation was observed for ^213^Bi-C595 at 48 hours, whereas significant morphological changes in cell clusters were not observed for C595 alone The variation in response of individual spheroids treated with the MAb C595 alone was minimal (P > 0.05); ^213^BiI_5_ and the non-specific control ^213^Bi-A2 did not exhibit apoptotic morphology, but the size and volume of the spheroids were reduced.

#### 4.2.1. Apoptosis

**Figure 4.**
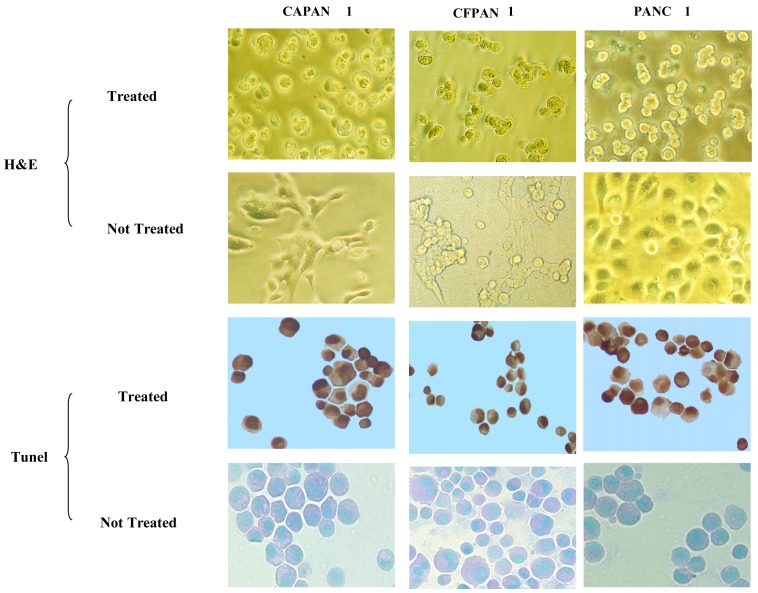
Apoptosis induced by C595-AC for pancreatic cancer cell lines [16].

A representative experiment is shown in Figure 4. Incubation of pancreatic cancer cells in C595-cDTPA-^213^Bi (A) causes morphological changes of treated cancer cells and induces apoptosis. The percentages of apoptotic cells are 73 ± 3 (CAPAN-1), 78 ± 2 (PANC-1) and 81 ± 3% (CFPAC-1) at 48 h after treatment with 370 kBq/10^4^ cells. However, the percentage of apoptotic cells for nonspecific control groups (B) is much lower (12 ± 3%) and apoptosis was not observed for medium only cells (C). Thus the major cause of cell death is apoptosis.

**Figure 5.**
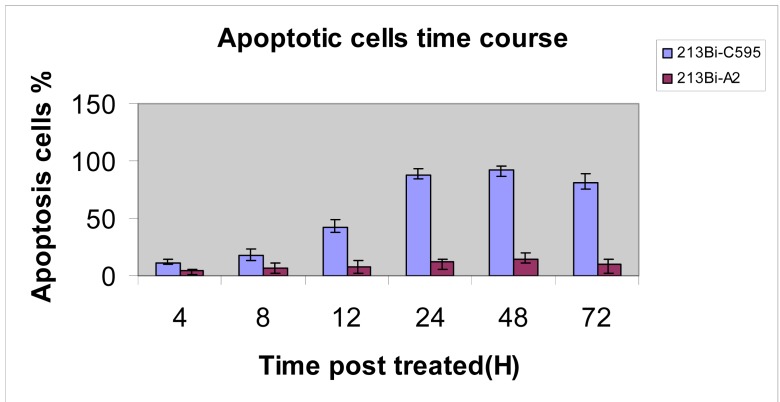
Time course for apoptosis for C595-AC compared with control [16].

The time course for apoptosis was determined and is shown in Figure 5, peaking at 24–72 h. Results are compared with the non-specific A2-AC, which is a measure of the background effect.

### 4.3. Maximum Tolerance Dose

#### 4.3.1. C595

**Figure 6.**
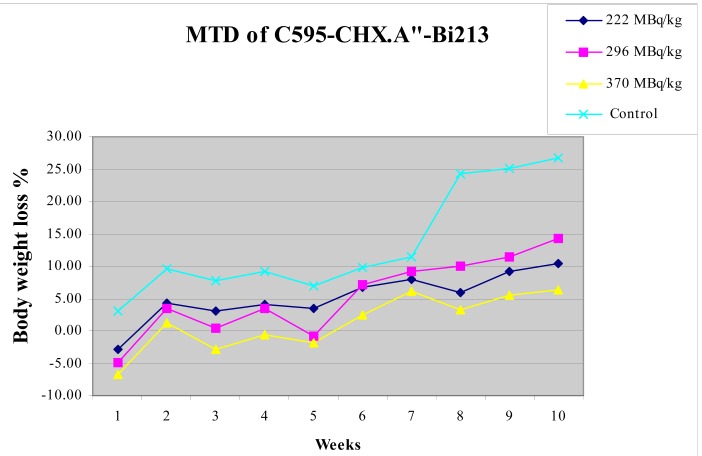
Dose effect of ^213^Bi-CHX-A″-C595 on body weight of mice over time [17].

Change in mouse bodyweight with time is shown in Figure 6 for IP injections of 222, 296 and 370 MBq/kg of AIC, using the CHX-A″ chelator. Test mice suffered a small, short-term weight loss before embarking on a growth rate similar to that for the controls over ten weeks.

The maximum tolerance dose for ^213^Bi-C595 for weight loss was found to exceed 370 MBq/kg. The maximum short-term body weight loss is 8% at ∼ one week in the high dose group (370 MBq/kg). Growth rates for treated mice were independent of injected activity and were similar to controls from 2 to 7 weeks after which the treated mice showed reduced growth rates.

#### 4.3.2. PAI2

Both cDTPA and CHX-A″ were coupled to PAI2 for labeling [49]. There was no significant difference in renal accumulation between these two chelators for up to 4 h (5 half-lives of Bi-213).

**Figure 7.**
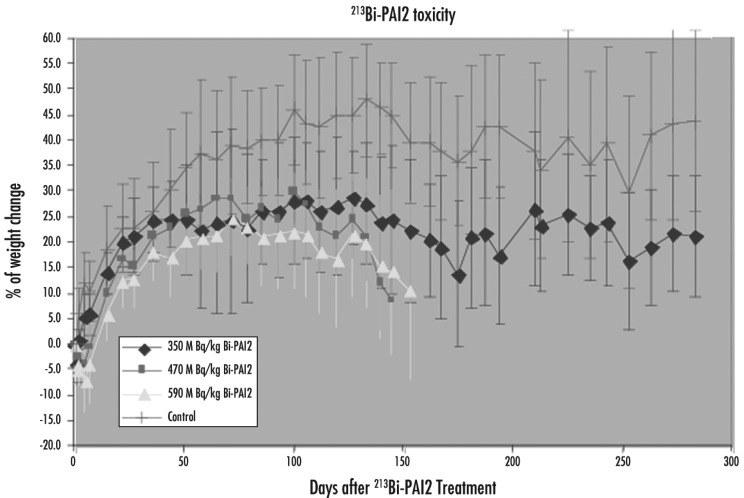
Maximum tolerance dose for ^213^Bi-PAI2 in mice. Only the 350 MBq/kg group maintained a steady bodyweight [49].

The survival rates of experimental mice in different dose groups are shown in Figure 7. The median time to end point after ^213^Bi-CHX-A″-PAI2 injection was 175 days for the 470 MBq/kg group, 162 days for 590 MBq/kg, (p > 0.05) whereas the control and 350 MBq/kg groups did not reach the end points. The endpoint rates were significantly different between the 350 MBq/kg and other treated mice (p < 0.05), while the difference was not significant between 350 MBq/kg and control mice. While hematology was normal at the end point, reduction in renal function was found, manifested by a severe increase in blood urea nitrogen (45–80 mmol/L, normal range 2.1–3.5 mmol/L).

**Figure 8.**
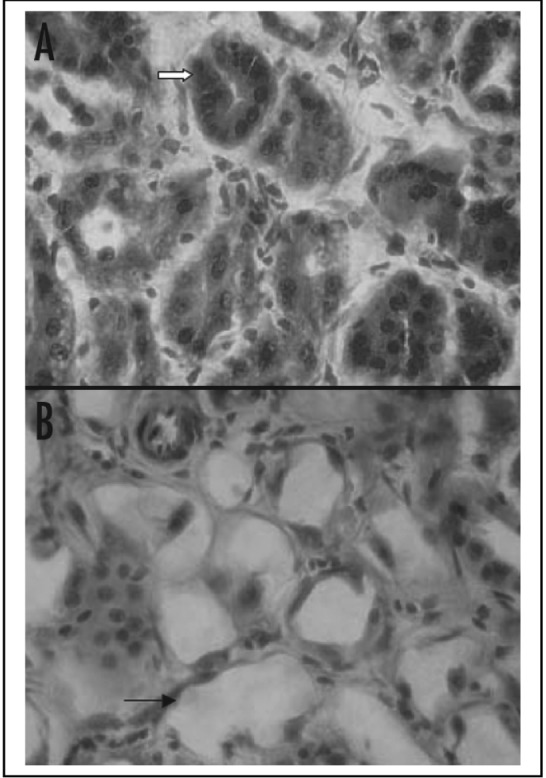
The normal renal structure in A is compared with radiation necrosis in B caused by ^213^Bi-PAI2 at 13 weeks post-injection. Arrow in image A indicates normal tubule compared with B which shows the tubular vacuolization and dilation, a result of the toxic effect. H&E staining with magnification of × 1000 [49].

Evidence of severe and widespread renal tubular necrosis was found in rabbit studies (Song 2007). Figure 8 compares normal renal structure with radiation necrosis caused by 350 MBq/kg of ^213^Bi-PAI2 at 13 weeks post-injection. The alpha radiation causes tubular vacuolization and dilation.

### 4.4. In Vivo Regression of CFPAC-1 Pancreatic Cancer by Local TAT

#### 4.4.1. C595

The subcutaneous tumor models used in these experiments have the advantage that local injections of AIC and tumor measurements are easy to perform. The local AIC injection was made at two days post-inoculation. Complete inhibition of tumor growth was observed for 3.7 MBq and above, one of five tumors (1/5) grew at 1.85 MBq while 5/5 tumors grew for both the nonspecific AIC group and cold C595 control mix group after 16 weeks.

Tumor volume reached 15 mm^3^ at 22 days for one out of five mice in the 1.85 MBq group, compared with an average of 13 days for all mice in non-specific 213Bi-A2 group and eight days for all mice in the cold C595 control.

The time to reach a tumor volume of 15 mm3 was significantly different between treatment and control groups (P < 0.001), while there were no significant differences for tumor growth times for the all treatment groups (P = 0.65). Cold C595 and ^213^Bi-A2 groups were significantly different; indicating that nonspecific Bi-213 has an effect (P = 0.004). Tumor growth was partially regressed by 1.85 MBq with tumor growth in one mouse delayed for approximate three weeks. Complete inhibition was observed for 3.7 MBq and above. Mice that received 1.85–7.4 MBq C595-AC had a median time to end-point of 112 days, which was significantly different to that for the cold C595 group (42 days) and 7.4 MBq ^213^Bi-A2 group (74 days) (P < 0.001).

### 4.5. In Vivo Regression of CFPAC-1 Pancreatic Cancer by Systemic TAT

#### 4.5.1. C595

**Figure 9.**
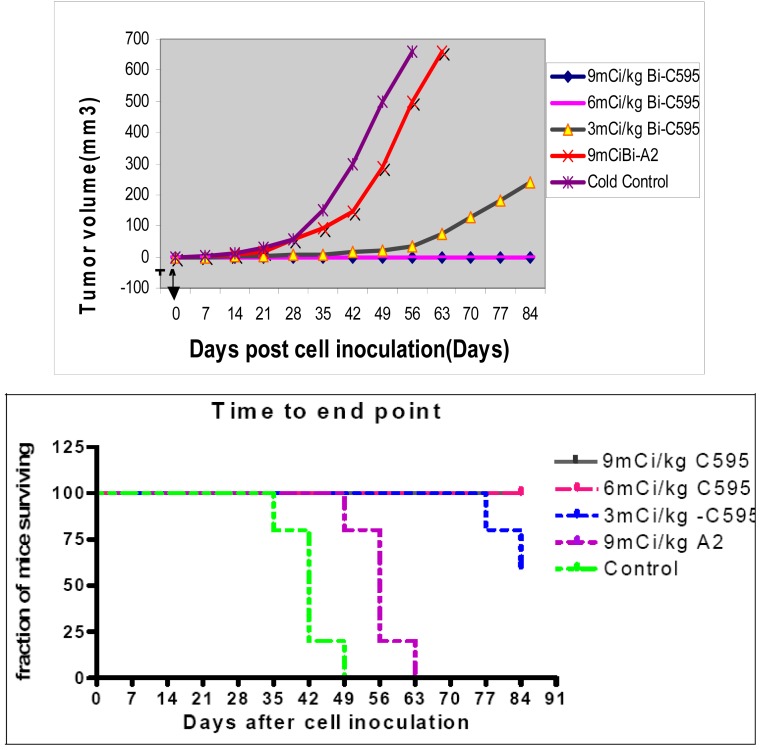
Systemic TAT with C595.[17] (**A**) Tumor growth effect after systemic ^213^Bi-C595 therapy; (**B**) Survival to endpoint for systemic therapy with Bi-213-C595 therapy.

The subcutaneous tumor model is the same as that for local TAT. A systemic (intraperitoneal) AIC injection of 111, 222 and 333 MBq/kg was made at two days post-inoculation of cancer cells. Complete inhibition of tumor growth (0/5) was observed for the 222 MBq/kg and above groups, 2/5 tumors continued to grow for 111 MBq/kg group, while 5/5 of tumors grew for non-specific AIC and cold control mix groups by 16 weeks. These results are shown in Figure 9A. Median post-inoculation time to the prescribed end point 112 days for 111, 222 and 333 MBq/kg group (P < 0.001); P value relative to the cold C595 control mix of 42 days and non-specific AIC control of 56 days, as shown in Figure 9B.

#### 4.5.2. PAI2

Less efficacious results were found for the PAI2-AC [18] than for the C595-AC, as 100% survival occurred at 9 mCi/kg (330 MBq/kg) for PAI2-AC and at 6 mCi/kg (220 MBq/kg) for C595-AC.

## 5. Discussion

### 5.1. C595

MAb-based therapeutics offers an important option for targeted control of metastases and to improve survival rate of patients with pancreatic cancer. These strategies include combination with cytotoxic drugs, conjugation with radionuclides or immunological effector cells. High specificity and affinity to targeted cancer tissue are essential for the selection of targeted antigens and targeting vectors. MUC1 is of special interest as upregulation is significantly correlated to the depth of invasion, lymph node metastasis, and peritoneal dissemination [50].

In our previous study we found that over 90% of primary pancreatic tumors expressed MUC1 while 95% of normal pancreas tissues did not [16]. Further, MUC1 expression was found on the surface of CFPAC-1, PANC-1 and CAPAN-1 cancer cells. The lethal pathway for the three cell lines *in vitro* after TAT was found to be predominantly by apoptosis.

The *in vitro* monolayer cell model yielded valuable information regarding the mechanisms of malignant growth, but was unsuitable in representing *in vivo* tumors, because the solid tumors grow in a three dimensional spatial array and the cells in these tumors are exposed to non-uniform distribution of oxygen and nutrients as well as other physical and chemical stresses. Because of the micro-environmental variations present, significant cellular heterogeneity may result.

Tumor spheroids represent a realistic three-dimensional growth and organization of solid tumors and, consequently, simulate much more precisely the cell-cell interactions and micro-environmental conditions found in these tumours.

In the present study, we tested MUC1 expression in cell clusters and in a nude mouse xenograft model, and found medium to strong tumor expression for all three cell lines, indicating that cancer cells do not lose MUC1 expression in passing from *in vitro* cell clusters to an *in vivo* animal xenograft.

The most effective radiation treatments are those that not only hit the intended target but also cause the greatest amount of lethal or non-repairable damage to DNA. Therefore, alpha particles are most effective in this respect. A large number of *in vitro* and *in vivo* experiments with alpha-immunotherapy have shown dramatic superiority over beta-immunotherapy, as only a few alpha hits of the nucleus are needed to achieved 63% cell kill [6,7,24-26]. Electron micrographs in studies of AIC treated lymphoma cells have demonstrated bizarre blebbing patterns, condensation of chromosomal material, and inter-nucleosomal DNA fragmentation patterns characteristic of programmed cell death (apoptosis), indicating that alpha particles may kill cells by apoptotic mechanisms.

Typical morphologic changes and a high percentage of TUNEL-positive cells in three pancreatic cancer cell lines were observed after treatment using ^213^Bi-C595 *in vitro*.

^213^Bi-C595 can target surface cancer cells, but killing internal cells may be different because of the short half-life of ^213^Bi and slow penetration of the antibody. Our results indicate that ^213^Bi-C595 is effective for pancreatic cancer spheroids up to 100 μm in diameter, and that targeting efficacy is in accordance with the expression of MUC1 in three cancer cell lines. The higher percentage of TUNEL positive cells was found in CFPAC-1 and PANC-1 spheroids because of high expression of MUC1 antigens while CAPAN-1 spheroids, having a lower response to MAb C595, also give the lowest fraction of apoptosis cells. These data indicate that the lethal pathway after TAT involves apoptosis. The size and volume reduction after nonspecific ^213^BiI4 and ^213^Bi-A2 was limited, and after 5–7 days, these spheroids regrew.

Therapeutic experiments were designed to evaluate the anticancer activity of ^213^Bi-C595 after administration at two days-post cancer cell inoculation and to optimize the dosage regimen. The two day xenograft model can mimic preangiogenic cancer cell clusters in the clinical condition because tumor blood vasculature has not formed at that time [51]. A single local injection of ^213^Bi-C595 at a dose of 1.85 MBq (74 MBq kg-1) or a single IP injection of ^213^Bi-C595 at a dose of 222 MBq kg-1 can completely suppress tumor growth over 16 weeks, while all control animals grew tumors. The growth inhibition of tumors and metastases was dose dependent. This means that ^213^Bi-C595 can control tumorigenesis by local or systemic TAT. Systemic TAT requires higher activity than local TAT to achieve comparable efficacy, because the AIC can easily diffuse to the cancer cells after local administration. Systemic therapy efficacy may depend on

1. vascular supply to the inoculation site,
2. physical half-life of the alpha emitting radioisotope,
3. the penetration rate of protein as a targeting vector,
4. dilution of dose by the blood volume.

These results indicate that ^213^Bi-C595 can inhibit growth of pancreatic cell clusters and preangiogenic lesions *in vivo*. Furthermore, these findings indicate that ^213^Bi-C595 can target and kill cancer micrometastases, *i.e.*, cells in transit or at the preangiogenic stage. Therefore, multiple metastatic sites at the minimal residual disease stage should be considered to be the most suitable targets for ^213^Bi-C595. Such a treatment may have a role as adjuvant therapy immediately after resection of macroscopic tumor to prevent early recurrence.

### 5.2. uPA

Elevated levels of the serine protease uPA, its receptor (uPAR), and inhibitor (PAI2), in tumor tissue emphasize their fundamental role in tumor invasion and metastasis and provide the rationale for this novel therapeutic strategy. The present system relies on the highly cytotoxic alpha emitting radioisotope ^213^Bi coupled to PAI2 to form a stable conjugate so as to kill uPA/uPAR-positive cancer cells. The short range of the alpha particle means a much greater fraction of the total energy is deposited in cells and very few nuclear hits are required to kill a cell.

The expression of uPA/uPAR has been found on a high percentage of human pancreatic cancers. Our results confirmed that uPA/uPAR is strongly expressed (+++) by the CFPAC-1 and PANC-1 cell lines, with medium level (++) expression in CAPAN-1 cell line. CFPAC-1 and CAPAN-1 are metastatic pancreatic cancer cell lines while PANC-1 is a primary pancreatic cancer cell line. Patients with uPA-positive tumor sections also showed over expression of uPA in lymph node metastases. These results indicate that cancer clones that escape from primary tumors do not lose uPA expression. We have also shown that CFPAC-1, CAPAN-1 and PANC-1 mice tumor xenografts strongly express uPA, indicating that cancer cells do not lose uPA expression from *in vitro* cell culture to *in vivo* animal model.

Using flow cytometry and confocal microscopy, we confirmed that uPA/uPAR was expressed on the surface of three pancreatic cancer cell lines. Our results indicate that membrane-bound uPA is an effective tumor surface marker for targeting pancreatic carcinoma. The cytotoxicity of ^213^Bi-PAI2 for pancreatic cancer cells *in vitro* proved to be receptor-specific, receptor intensity- and activity-dependent. Compared with the non-specific control, ^213^Bi-PAI2 exhibits high levels of receptor-selective cytotoxicity, requiring very low activity (D_0_= 133–185 kBq) to kill high cell concentrations (2 × 10^4^ cells/300 μL) for three cell lines, whereas the D_0_ value for the non-specific control (^213^Bi-BSA) and ^213^BiI3 was found to be 12–16 times higher for uPA-positive cells. CFPAC-1 cells with the highest intensity of surface uPA/uPAR expression were found to have the lowest D_0_ value, compared to PANC-1 and CAPAN-1 cells, respectively. These results indicate the specificity of the AC for cell expression of different levels of uPA. Therefore, it is clear that ^213^Bi-PAI2 is an effective and specific radio-labeled agent for monolayer-cultured cells *in vitro*.

At a specific activity of 3 mCi/mg of ^213^Bi-cDTPA-PAI2, we have ∼ 3 × 10^5^ cold PAI2 molecules per hot PAI2. If there are 10^6^ receptors per cell, with high binding affinity and an excess of ^213^Bi-PAI2 molecules, then there could be up to 30 atoms of Bi-213 per cell, which would give about six nuclear traverses and minimal survival [39]. However, the actual uPAR expression on the pancreatic cell lines has not been measured. The 50% internalization time for alpha-PAI2 has been measured at 8.4 min in breast cancer cells [37]. As the cell kill efficiency depends on the location of the alpha emitter, rapid internalization would enhance the cytotoxicity by a factor of two or more. Thus the results reported by Macklis [39] could be achieved in these alpha-PAI2 experiments. Indeed, the key observation is that cytotoxicity is achieved under the given experimental conditions.

## 6. Clinical trials

### 6.1. C595 vs. PAI2

Both vectors target receptors with similar expression in pancreatic tumor sections, the majority of sections showing a strong 2+ to 3+ relationship. There is an element of complementarity, but as shown in Figure 3, the expression of receptors is correlated. The advantage of the low MW PAI2 (MW = 47 kDa) is that tumor capillary permeability might be expected to be higher than for the C595 antibody. However, this potential gain (not yet proven) may be offset by the higher renal uptake of PAI2 and subsequent higher radiation nephritis arising from alpha emission.

One problem already experienced with a Human Research Ethics Committee relates to the poor life expectancy of end stage pancreatic cancer patients in a Phase I trial, such that long term effects will not be observed if patient prognosis does not change. As apparent from this paper, both the alpha-PAI2 and alpha-C595 conjugates are ready for a Phase I trial.

### 6.2. Tumour Antivascular Alpha Therapy (TAVAT)

In the Phase I clinical trial of targeted alpha therapy for metastatic melanoma [14] tumor regressions were unexpectedly observed with 10% of 38 subjects experiencing partial tumor regression and 40% stable disease. This effect was not expected because the short half life (46 minutes) of Bi-213 and short alpha range (80 μm) would preclude effective diffusion of the AC through the tumors and achieving an average cytotoxic tumor dose.

Thus tumor antivascular alpha therapy was proposed, such that extravazation of the AC would lead to saturation of antigens expressed by the contiguous cancer cells [15]. While alpha emissions would occur in all directions, there would be a concentration of dose back into the capillary causing endothelial cell depth and closure of capillaries. If enough capillaries are so affected, the tumor is starved of oxygen and nutrients and regresses. The clinical effect of TAVAT is expected to also manifest in pancreatic cancer tumors, causing tumor regression via oxygen deprivation.

## 7. Conclusions

A strong case is presented for the potential success of targeted alpha therapy for the management of pancreatic cancer. Both uPA and MUC1 have similar expression in pancreatic cancer tumors and the alpha conjugates of c595 and PAI2 have similar *in vivo* efficacy. The TAVAT principle means that while both could be effective in regressing tumors, the smaller PAI2 molecule could be more effective in achieving perivascular invasion. However, this unproven advantage may be more than offset by higher radiation doses arising from the PAI2 conjugate being filtered by the kidneys.

## Figures and Tables

**Table 1.** Expression of uPAR and MUC1 in cell lines and tumor tissues.

	**uPAR**	**MUC1**
**Reference**	[17]	[16]
**Cell lines**	3/3 +++	1/3 ++ 2/3 +++
**Xenografts**	1/3 ++ 2/3 +++	1/3 ++ 2/3 +++
**Tumors**	4/30 - 3/30 + 12/30 ++ 11/30 +++	5/53 - 5/53 + 15/53 ++ 28/53 +++
**Lymph nodes**	2/6 ++ 4/6 +++	
**Normal pancreas**	10/10 -	3/53 + 50/53 -

**Table 2.** Comparison of cell line properties.

**Vector**	**Cell line**	**ICC**	**Flow**#	**Confocal**	Do* kBq
C595:MUC1	Capan-1	++	40	++	170
	Cfpac-1	+++	500	+++	140
	Panc-1	+++	80	+++	160
#394:uPA	Capan-1	++	88	++	190
	Cfpac-1	+++	92	+++	130
	Panc-1	+++	91	+++	170
2200-2500	all cells				2200-2500

# median value;

* D_0_ = 37% cell survival (kBq/300 μL);

ICC: immunocytochemistry
